# Interoceptive signals and emotional states shape temporal perception through heart rate modulation

**DOI:** 10.3389/fpsyg.2025.1610347

**Published:** 2025-07-03

**Authors:** Maria Volodina, Anna Rusinova, Kristina Terenteva, Vladimir Kosonogov

**Affiliations:** ^1^Center for Bioelectric Interfaces, Institute for Cognitive Neuroscience, Higher School of Economics University, Moscow, Russia; ^2^Institute of Neuroscience and Neurotechnologies, Federal State Autonomous Educational Institution of Higher Education “N.I. Pirogov Russian National Research Medical University”, Ministry of Health, Moscow, Russia; ^3^Institute for Cognitive Neuroscience, Higher School of Economics University, Moscow, Russia; ^4^Affective Psychophysiology Laboratory, Institute of Health Psychology, Higher School of Economics University, St. Petersburg, Russia

**Keywords:** time perception, emotions, interoception, awareness, heart rate

## Abstract

**Background:**

This study investigated the relationships between interoceptive signals, emotions, and time perception, with a particular focus on the mediating role of heart rate (HR). Emotional video stimuli were used to elicit specific emotional responses, while participants’ attentional focus was manipulated (internal vs. external) to examine its influence on temporal judgments.

**Methods:**

We tested several hypotheses using a combination of linear mixed models and Bayesian mediation analysis. Emotional content, heart rate, attentional focus, and interoceptive accuracy were analyzed for their effects on time perception. Participants viewed 36 video clips designed to elicit positive, negative, or neutral emotions, and their duration estimation errors, normalized heart rates, and subjective ratings were recorded.

**Results:**

Emotional content significantly influenced time perception. Negative and neutral videos were underestimated in duration, while positive videos showed smaller estimation errors. Heart rate partially mediated the effect of emotions on time perception, with slower heart rates linked to greater underestimation of durations. However, this mediating effect was smaller compared to other potential mechanisms not explored in this study. Contrary to prior research, no correlation was found between interoceptive accuracy and time perception precision, likely due to methodological differences in task design and measures of interoception. An internal focus of attention was associated with greater underestimation of time and lower normalized heart rate. However, no significant interaction was observed between attentional focus, heart rate, and emotional valence.

**Conclusion:**

These findings highlight the multifaceted nature of time perception, emphasizing the role of both physiological processes and subconscious interoceptive signals. The partial mediation of heart rate underscores its importance in shaping temporal judgments, while the lack of interaction with attentional focus suggests that these effects may be driven by unconscious mechanisms. The results contribute to a deeper understanding of how emotions and bodily signals interact to shape time perception and underscore the need for further research into individual differences and unconscious influences on temporal judgments.

## Introduction

The perception of time is crucial to human existence and our daily actions. Our ability to understand and measure time is essential for planning, coordinating, and performing our activities. Without an adequate perception of time, we would struggle to organize our tasks, interact with others, and adapt to changes in our environment and navigate and function effectively in the world. Temporal distortions are frequently reported in psychiatric and neurological disorders, including depression (time slowing), anxiety (time speeding), ADHD (irregular timing), and schizophrenia (fragmented or disjointed temporal flow) (Stanghellini et al., 2016; Ren et al., 2023; Holman et al., 2023; Ptacek et al., 2019). This can manifest as a loss of the immediate flow of time, making events feel isolated and unrelated, which contributes to difficulties in organizing daily activities and maintaining social interactions. Thus, the study of time perception is not only fundamental to understanding human cognition, but also holds significant potential for practical applications that can positively impact individual and societal well-being and have practical significance for the diagnosis and treatment of various psychiatric and neurological disorders.

There are many different factors and mechanisms that can affect our perception of time, and emotional states are among the most influential. Emotions shape our experience of time by engaging internal timing mechanisms in response to personally significant or evolutionarily relevant events (Droit-Volet and Gil, 2009). They can lead to either overestimation or underestimation of durations, making time appear to slow down or speed up depending on the context. For instance, negative emotional stimuli are frequently associated with a lengthening of perceived time, which is often explained by an acceleration of the internal clock (Yamada and Kawabe, 2011). Our subjective well-being also strongly affects how we perceive time. Time seems to speed up during pleasurable activities but drags on during periods of boredom (Wittmann, 2009). Social isolation, and stress can slow the perception of time, while high levels of social satisfaction and low levels of stress can, conversely, speed up its passage (Droit-Volet et al., 2020; Martinelli et al., 2020). However, an important question in this field concerns whether these distortions are driven primarily by the valence of the emotion (positive or negative) or by the level of arousal it elicits. There is evidence that overestimation of time occurs at a moderate level of arousal for positive and negative valences, but at the same time underestimation also occurs in a state of high arousal with a negative valence (Van Volkinburg and Balsam, 2014). A substantial body of evidence points to arousal as the primary driver of time distortions. For example, Fayolle and Droit-Volet (2014) found that high-arousal emotion such as anger significantly affected time judgments, while low-arousal negative emotions like sadness did not—despite their similar valence. According to pacemaker-accumulator models of internal timing, increased arousal accelerates the rate of the internal pacemaker, leading to the perception that more time has passed (Gibbon, 1977). In sum, the perception of time is shaped by a dynamic interplay between emotional valence and physiological arousal, with growing evidence suggesting that arousal exerts a more direct influence on the internal mechanisms of time estimation. Nevertheless, debate persists over whether emotions independently distort temporal experience or whether these effects are fully mediated by their arousal component.

The question of how we perceive time intervals has long existed. There are several hypotheses regarding the physiological basis of time perception. According to the internal clock model (Gibbon, 1977), and the attentional gate model (Block and Zakay, 1997), the basic element of each time estimation process is a pacemaker, producing pulses at a certain rate, which are accumulated and stored in working memory. Comparing the number of accumulated pulses with the number of reference durations from previous learning processes yields a duration judgment (Gibbon et al., 1984).

Growing recognition of the body’s role in shaping temporal experience has led researchers to consider physiological and interoceptive mechanisms as integral components of time perception. Craig’s (2009) embodiment concept suggests a direct link between time perception and bodily processes. It postulates that our sense of time is connected to visceral processes because they share common neural substrates, such as the insular, medial prefrontal, and orbitofrontal cortices (Craig, 2003). The heart is a strong candidate for the role of the internal clock because it produces a consistent rhythm. Its rhythmic activity not only regulates blood flow, but also sends continuous signals to the brain. These signals are processed by interoceptive centers, which help integrate bodily rhythms with our perception of time.

Research into the effect of heart rate on the perception of time has yielded mixed and sometimes contradictory results. While some studies found no impact of heart rate on time estimation, others discovered significant correlations. To begin with, Hawkes et al. (1961) found that rhythmic stimulation could change the subjective perception of time intervals (Hawkes et al., 1961). This study used various drugs to alter participants’ physiological parameters and found a positive correlation between heart rate and estimated time. More recent study examined the effect of heart rate on time estimation in divers, cyclists, and recreational athletes, finding significant correlations between time estimates and heart rate (Jamin et al., 2004). It has also been hypothesized that the activity of the sympathetic and parasympathetic nervous systems may influence time perception. One study found that increased activity of the sympathetic system, indicated by higher heart rate and increased skin conductance response frequency, was associated with faster time perception (Ogden et al., 2022). However, some studies have shown that time perception might not be related to physiological cues like the heartbeat but is instead determined by external sensory stimuli and cognitive processes (Fontes et al., 2016).

Evidence supporting the role of interoceptive signals in time perception comes from studies that explore how attentional focus affects the relationship between emotions and time judgments. Specifically, an internal focus of attention has been found to amplify the effect of emotions on duration estimations (Pollatos et al., 2014). For instance, fear tends to lengthen perceived time, particularly when attention is directed inward, while amusement has the opposite effect, shortening perceived time. Emotions not only alter arousal and attentional states, but also interact with bodily awareness to shape temporal judgments. Pollatos et al. (2007) demonstrated that individuals with higher interoceptive accuracy exhibit stronger heart rate responses to emotional stimuli and rate them as more arousing, suggesting that bodily signal perception intensifies emotional processing (Pollatos et al., 2007). Similarly, Özoğlu and Thomaschke (2020) found that while emotional arousal generally causes temporal overestimation, individuals with higher interoceptive accuracy show a reduced susceptibility to this distortion. However, despite these advances, the mechanisms through which interoception interacts with emotional arousal to shape temporal judgments remain incompletely understood and require further empirical investigation using multimodal physiological measures.

The present study aimed to explore the complex interplay between emotions, physiological processes, and time perception, with the overarching hypothesis that interoceptive signals, particularly heart rate, mediate the influence of emotions on time estimation. To investigate this central hypothesis, we formulated several specific predictions. First, we hypothesized that emotional valence significantly influences time perception. Consistent with Pollatos et al.’s (2014) results, we predicted that negative emotions would lead to an overestimation of perceived time. Second, we hypothesized that variations in heart rate would be associated with changes in temporal judgments, predicting that higher heart rate would result in subjective time overestimation (Lake et al., 2016). Third, we proposed that heart rate serves as a mediator in the relationship between emotional states and time perception, suggesting a physiological pathway through which emotions impact temporal judgments. Fourth, we examined whether interoceptive accuracy is positively associated with the precision of time perception, hypothesizing that individuals with heightened sensitivity to internal bodily signals demonstrate more accurate temporal processing. Finally, to examine the modulation of attention to interoceptive signals, we manipulated focus of attention by directing participants toward either an internal or external focus. We investigated whether an internal focus of attention amplifies the effects of emotions on time perception, thereby modulating the strength of this relationship.

Time perception is commonly investigated using several well-established behavioral paradigms, each engaging different cognitive processes and therefore potentially yielding distinct patterns of results depending on task demands and individual differences. In estimation tasks, participants are asked to judge the duration of a presented stimulus, often on a continuous scale (Angrilli et al., 1997). In reproduction tasks, individuals experience a time interval and are then required to reproduce it by pressing a button to indicate when they believe an equivalent amount of time has passed, in some research also using supra-second intervals (Osato et al., 1995; Teghil et al., 2020). Production tasks involve instructing participants to generate a specific time interval, assessing their ability to internally produce temporal durations (Coull et al., 2013). In bisection tasks, participants performed the task of distinguishing time intervals (Dormal et al., 2018; Mioni et al., 2016), with the latter focusing on sub-second intervals ranging from 450 to 750 ms. The cognitive and physiological mechanisms underlying time perception differ depending on interval length, while sub-second timing is often considered to reflect automatic, sensorimotor processes, supra-second timing is thought to involve higher-order mechanisms including working memory, attention, and interoceptive processing (Meissner and Wittmann, 2011). The choice of paradigm can significantly influence observed effects of emotional and interoceptive factors on timing. Some tasks rely more heavily on working memory or attentional control, while others may better capture the contribution of bodily signals to temporal perception.

We considered that the duration estimation task in the supra-second time range (40–60 s) was the most appropriate choice for our study, as it aligns closely with our research aims and the theoretical framework linking emotional experience, interoceptive signals, and subjective time perception. First, our primary research question centers on the role of interoceptive signals (particularly heart rate) in mediating the effects of emotion on temporal judgments. Supra-second intervals are particularly well-suited for studying such interactions, as this duration range is known to engage higher-order cognitive processes, including working memory, sustained attention, and interoceptive awareness (Meissner and Wittmann, 2011). Second, the estimation task, in contrast to reproduction or production tasks, does not require a motor response to demarcate time (such as pressing a button after a perceived interval). This minimizes confounding effects of motor timing variability and shifts the focus toward the subjective experience of time, which is central to our investigation. It also makes the task more naturalistic and cognitively oriented, which is relevant when assessing the impact of emotional engagement and internal bodily cues. Third, by using extended stimulus durations (20–60 s), we created conditions that require sustained emotional engagement and allow sufficient time for physiological responses, such as heart rate changes, to manifest and stabilize.

## Methods

### Participants

The sample consisted of 40 participants ranging in age from 18 to 37 years, with thirteen men and twenty seven women and the mean age of 22.4 (SD = 6.89).

The initial sample size was estimated based on prior findings on the interaction between attentional focus and emotion in time perception reported in Pollatos et al. (2014), whose design we adapted and extended. A required sample size of 42 participants was projected to detect comparable effects. *Post hoc* power analysis based on our data for key associations between heart rate and time perception indicated a statistical power of approximately 94%.

Inclusion criteria were the following: participants were selected from individuals aged 18 to 40 years, participants should be without diagnosed mental illnesses or brain disorders and not taking drugs that affect the central nervous system, such as antidepressants or sedatives. Exclusion criteria were the participant’s inability to complete the experiment and the incompatibility of data due to specific physiological parameters affecting data acquisition. Two participants were excluded from the analysis for the following reasons: one due to technical issues with the ECG signal during the heartbeat counting task, which prevented reliable peak detection and calculation of interoceptive accuracy; the second participant’s interoceptive accuracy score fell outside the acceptable range based on Tukey’s outlier test. Thus, the data from 38 individuals were included in the final analysis with twelve men and twenty six women and the mean age of 22.0 (SD = 6.58). The experiment was conducted in accordance with the Declaration of Helsinki and approved by Inter-University Surveys and Ethical Assessment of Empirical Research (#52). Participants received compensation (8.33 USD at purchasing power parity). All participants provided written informed consent.

### Stimuli

In the main phase of the experiment, participants were presented with 36 video stimuli selected from a pre-tested database (Kosonogov et al., 2025) to ensure they elicited distinct emotional responses. To achieve this, we included 12 videos that evoked negative emotions, 12 that evoked neutral emotions, and 12 that evoked positive emotions, as determined by prior ratings in the database. Since the original videos were 1 min in duration, they were edited to vary in length in order to investigate how participants estimated their duration. See Supplementary materials for pre-validated characteristics of the stimuli (Supplementary Table S1) and subjective ratings provided by participants in the present study (Supplementary Table S2).

The database was preliminarily validated by 185 Russian-speaking participants (74% female, mean age = 24.8 years, SD = 8.0). Each participant viewed and rated only one subsample (randomly assigned), while each video clip was assessed by 30 raters, who were randomly selected from the overall sample. Ratings were collected using a 9-point scale for both valence (1 = very unpleasant, 9 = very pleasant) and arousal (1 = very calm, 9 = very arousing).

Valence ratings of the video stimuli used in the present study were as follows (ranging from 1 for “very negative” to 9 for “very positive”): negative clips: M ± SD = 2.61 ± 0.16; positive clips: M ± SD = 7.38 ± 0.21; neutral clips: M ± SD = 4.89 ± 0.27.

Negative videos were rated as more negative (*t* = −24.78, *p* < 0.001, *d* = −10.56) than the neutral ones. Positive videos were rated as more positive (*t* = 25.07, *p* < 0.001, *d* = 10.69) than the neutral ones.

Ratings of arousal for the video stimuli used in the present study were obtained on a 9-point scale ranging from 1 (“very calm”) to 9 (“very arousing”). The mean arousal ratings were as follows: M ± SD = 5.96 ± 2.32 for negative clips, 6.02 ± 2.01 for positive clips, and 3.81 ± 1.96 for neutral clips.

Negative and positive videos were rated as more arousing than neutral ones (negative: *t* = 3.85, *p* < 0.001, *d* = 0.99; positive: *t* = 5.01, *p* < 0.001, *d* = 1.29), with no significant difference between them (*t* = −0.99, *p* = 0.326, *d* = −0.26).

Within each emotional category, we selected two videos for each of six predefined durations: 21, 28, 35, 42, 49, and 56 s. During the experiment, participants rated the emotional impact of the videos themselves, and these subjective ratings were subsequently used to categorize the videos into emotional categories (negative, neutral, or positive) for each participant individually. This approach ensured that the classification of videos was based on the specific emotional responses of each participant, rather than relying solely on the pre-existing database ratings.

### Procedure

**Figure 1:**
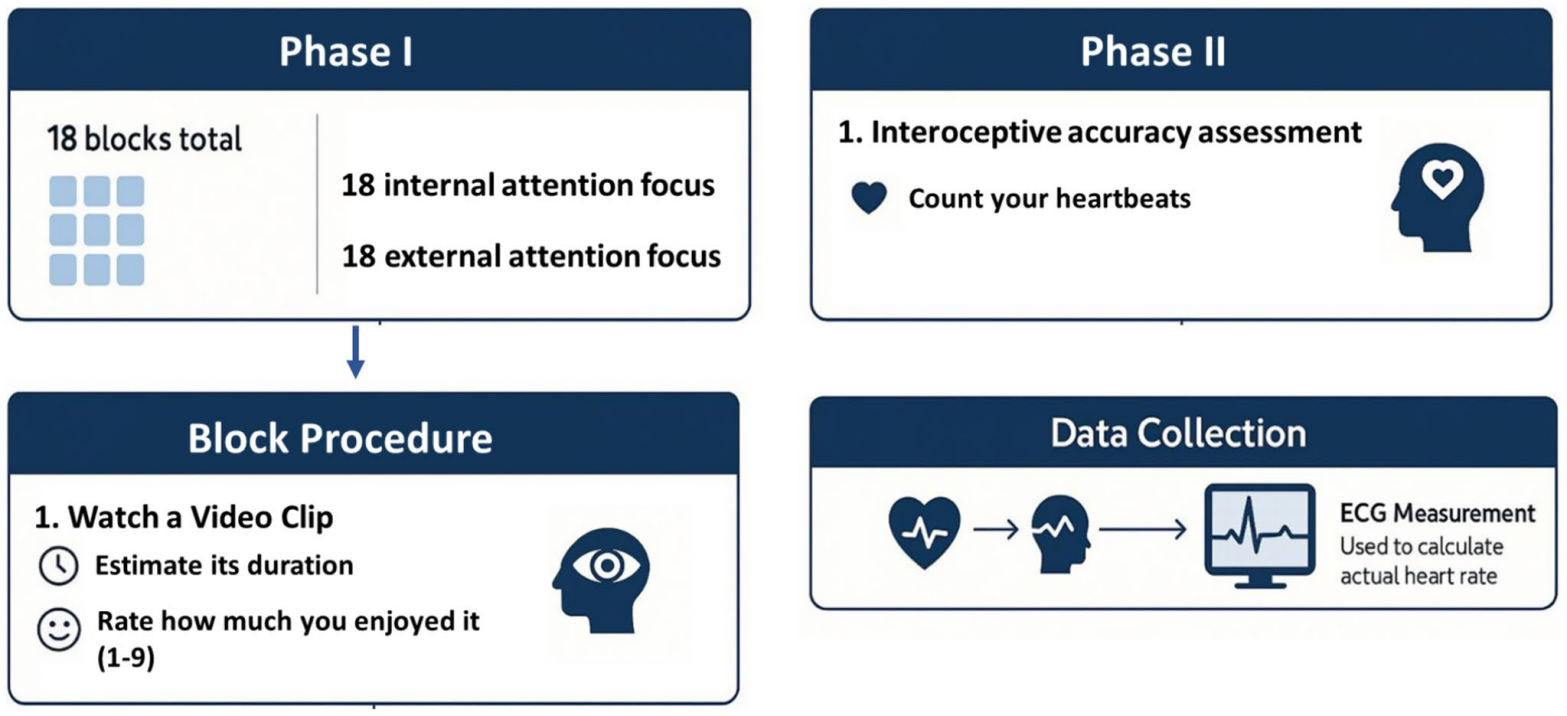
The design of the experiment. The sequence of internal and external attention focus varied among participants.

The design of the experiment is shown in Figure 1.

Participants were given three minutes to rest and minimize any activity. This resting phase aimed to stabilize their physiological performance before they were exposed to the experimental stimuli, allowing for a more accurate investigation of how visual content affects cognitive processes.

The experimental session was divided into two equal parts. In the first part, participants focused on the internal sensations triggered by the videos (internal attention focus). In the second part, they concentrated on the video’s details (external attention focus). The order of these conditions varied among participants. After watching each video, participants rated its duration in seconds and assessed their feelings about the content on a scale from 1 to 9, where 1 represented “very unpleasant” and 9 represented “very pleasant.” The final phase of the study involved a task designed to evaluate interoceptive accuracy.

Participants were instructed to count their heartbeats over six predefined time intervals: 21, 28, 35, 42, 49, and 56 s. This procedure was based on the heartbeat counting task described by Garfinkel et al. (2015), with minor modifications to better align the temporal structure of the task with the durations of the video stimuli used in the main experimental phase. The selected intervals corresponded to the specific time frames of the videos participants had previously viewed, ensuring greater consistency across tasks and minimizing potential differences in attentional engagement and temporal processing. To reduce the likelihood that participants would estimate the number of heartbeats based on their knowledge of standard heart rates, they were asked to count heartbeats during intervals of varying lengths, without being informed of the exact durations beforehand. Interoceptive accuracy was evaluated according to the following formula:


$$\begin{array}{l} 1/6\;\Sigma \;(1-(|\text{actual heartbeats}-\text{reported heartbeats}|\\ /\text{actual heartbeats}) \end{array}$$

Presentation of the stimulus material and recording of the subjects’ responses were performed using the Psychopy program (Peirce, 2007). ECG was continuously monitored throughout the experimental session.

### ECG recording

Throughout the experiment, ECG was recorded using an NVX52 amplifier (Medical Computer Systems, Russia) and NeoRec software (Medical Computer Systems, Russia) and digitized at a sampling rate of 250 Hz. Clamp ECG electrodes were positioned on the wrists and the ankle of the right foot. Synchronization of the ECG signal and stimulus presentation was performed using a photo sensor fixed in the upper right corner of the monitor.

### ECG data processing

The following filters were used: 0.5 Hz high-pass filter, 25 Hz low-pass Butterworth filter, notch filter at 50 Hz. R-peaks on the ECG were detected and instantaneous HR was calculated using the bio_process function from the NeuroKit library in Python (Makowski et al., 2021). For each video, the average HR was calculated, followed by standardization of HR and computation of the *z*-score based on the video-averaged HR values.

### Duration estimation error calculation

The estimation error of video duration was computed as a measure of the relative deviation between participants’ estimated durations and the actual durations of the stimuli. Specifically, the estimation error (estimation_error) was calculated using the following formula:


$$\begin{array}{l} \text{estimation}\_\text{error}=(\text{estimated duration}-\text{actual duration})\\ /\text{actual duration}. \end{array}$$

This formula provides a normalized measure of error, expressed as a proportion of the actual duration. Positive values indicate overestimation (participants perceived the duration as longer than it actually was), while negative values indicate underestimation (participants perceived the duration as shorter than it actually was).

Trials for which the estimation error fell outside the Tukey’s outlier bounds (i.e., below Q1 − 1.5 × IQR or above Q3 + 1.5 × IQR) were excluded from further analysis.

### Data analysis

Statistical analyses were conducted using Python scripts and Statistica 64 software. To examine how interoceptive signals, emotions, and time perception are related, we used the following protocol.

First, we employed linear mixed models (LMM) to examine the relationships between key variables: video pleasantness, heart rate (HR), duration estimation error, and focus of attention. LMMs allowed us to account for individual differences by incorporating participant ID as a random factor, providing a robust evaluation of overall associations while controlling for variability across participants. Four models were specified to test distinct hypotheses regarding the interplay between emotional experience, physiological arousal, and time perception. In Model 1, subjective video pleasantness (est_like, on a 9-point scale) was treated as the independent variable (IV), and duration estimation error (estimation_error) as the dependent variable (DV). Model 2 tested the association between normalized HR (*z*_score_HR) and estimation error, with *z*_score_HR serving as the IV and estimation_error as the DV. Model 3 examined how subjective pleasantness influenced HR, with est_like as the IV and *z*_score_HR as the DV. Finally, Model 4 explored whether the effect of HR on time perception varied depending on the focus of attention, testing an interaction between *z*_score_HR (IV) and focus (coded as internal or external), with estimation_error as the DV. The statsmodels.formula.api.mixedlm function in Python was used for all analyses, with random intercepts fitted for participants (subj_id).

Next, to gain detailed insights into specific pairwise comparisons between emotional conditions, we conducted analyses using estimated marginal means (EMMs). For this, videos were categorized based on participants’ subjective pleasantness ratings into three valence groups: negative (scores <4), neutral (scores 4–6), and positive (scores 7–9). The analysis was implemented using the lme4 package for model fitting and the emmeans package for *post hoc* comparisons in R (Lenth, 2020). Estimated marginal means were computed for each level of valence, and pairwise contrasts between valence conditions were evaluated with Tukey’s adjustment to control for multiple comparisons. All statistical analyses were performed via the rpy2 interface within Python.

To validate the absence of an interaction between focus of attention and normalized heart rate (HR) in their influence on duration estimation error, as suggested by the LMM results, we conducted a Bayesian hierarchical analysis using the PyMC library. This analysis incorporated random intercepts for individual participants to account for within-subject 38variability and estimated fixed effects for normalized HR, focus of attention, and their interaction. Focus of attention was coded as a binary variable (“internal” = 1, “external” = 0).

Finally, to test the hypothesis that heart rate mediates the effects of emotions on time perception, we performed a Bayesian mediation analysis using a hierarchical linear mixed-effects model. The model included two pathways: Path A (emotions → normalized HR) and Path B (normalized HR → time perception error). Random intercepts were included for participants to account for individual variability, and the indirect effect was calculated as the product of coefficients from Path A and Path B.

Pearson correlation analysis was used to assess the relationship between averaged across all subjects normalized HR and video pleasantness ratings, as well as between interoceptive accuracy of a particular subject and his/her average duration estimation error averaged across all stimuli. While the former served as a confirmatory step, the latter was limited by the sample size of 38 participants, which is sufficient to detect medium-to-strong correlations (|*r*| ≥ 0.34 for *α* = 0.05 and power = 0.80) but may overlook weaker effects.

For clarity and ease of interpretation, the results are presented not in the order in which the methods were applied, but rather in the logical sequence of exploring the interactions between different variables, starting from the influence of video pleasantness and heart rate to the role of interoceptive accuracy and focus of attention.

A more detailed description of the statistical models used in this study and the rationale for the chosen statistical approaches is provided in Supplementary Text S3.

## Results

### Video stimuli pleasantness and time perception

Initially, we explored how the pleasantness of the video impacted the participants’ perception of its duration. To achieve this, we examined the relationship between participants’ subjective ratings of video pleasantness and their accuracy in estimating video duration. A negative error indicated an underestimation of the video’s length, while a positive error reflected an overestimation.

The mixed linear model regression analysis with a random factor revealed that the subjective evaluation of pleasantness of video was significantly associated with estimation error of video duration, *β* = 0.013, SE = 0.000, *z*-score = 5.140, *p* < 0.001, 97.5% CI [0.008, 0.018]. The positive beta coefficient indicates that higher subjective pleasantness ratings of the video are associated with larger errors in estimating video duration. However, further analysis clarifies the nature of this relationship.

According to subjective ratings of video pleasantness, videos were labeled as negative (score less than 4 out of 9), neutral (4–6 points), and positive (7–9 points). Notably, pre-test ratings indicated comparable levels of arousal for videos categorized as negative and positive, suggesting that differences observed in the main experiment are not attributable to variations in emotional intensity along this dimension. More detailed statistics is provided in Supplementary Text S4.

**Figure 2:**
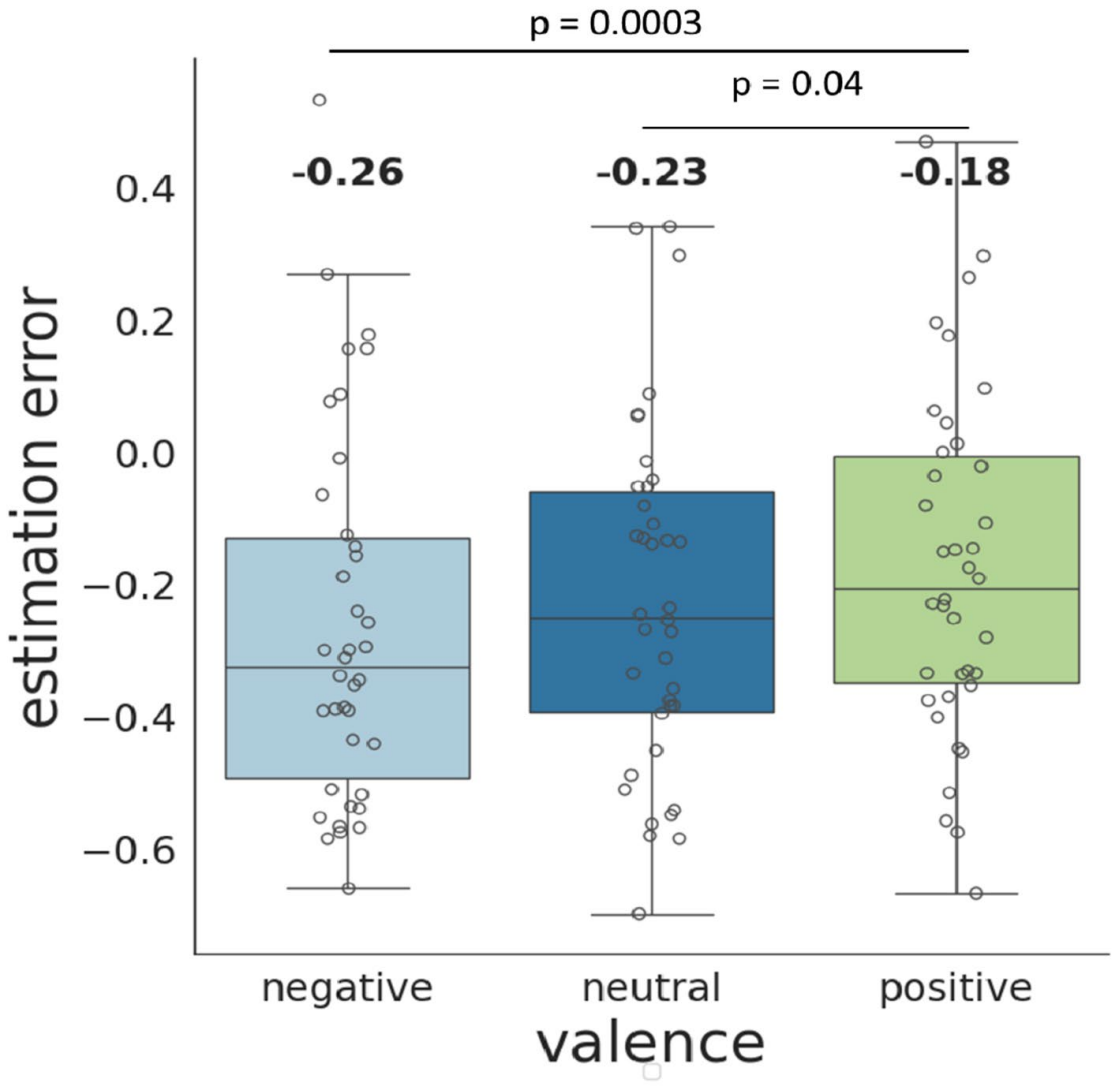
Error of video duration estimation when viewing negative, neutral, and positive videos. The box indicates the interquartile range (IQR), with the lower edge representing the 1st quartile (Q1) and the upper edge representing the 3rd quartile (Q3). The box contains the middle 50% of the data and medians, while the whiskers extend to the minimum and maximum values. Gray unfilled circles indicate individual data points. The indicated numbers represent the mean values for each group. The *p*-values for the *post*-*hoc* Tukey test are provided.

Estimated marginal means analysis revealed a significant difference in time estimation error between negative and positive emotional valence conditions (estimate = −0.079, SE = 0.016, *p* < 0.0001), as well as between neutral and positive conditions (estimate = −0.058, SE = 0.014, *p* = 0.0001). In both cases, durations were significantly underestimated in the negative and neutral conditions compared to the positive condition. No significant difference was observed between the negative and neutral valence conditions (estimate = −0.021, SE = 0.015, *p* = 0.337) (Figure 2).

Meanwhile, no interaction between the factors “video valence” and “focus of attention” was found.

### Video stimuli pleasantness and heart rate

Next, we examined the relationship between the perceived pleasantness of the video and the participants’ heart rate during its viewing. The pleasantness factor was significantly associated with normalized heart rate (*z*-score) [*β* = 0.043, SE = 0.014, *z*-score = 3.146, *p* = 0.002, 97.5% CI (0.016, 0.069)], with participant ID included as a random effect. The positive beta coefficient indicates that as the perceived pleasantness of the video increased, participants’ normalized heart rate also increased.

**Figure 3:**
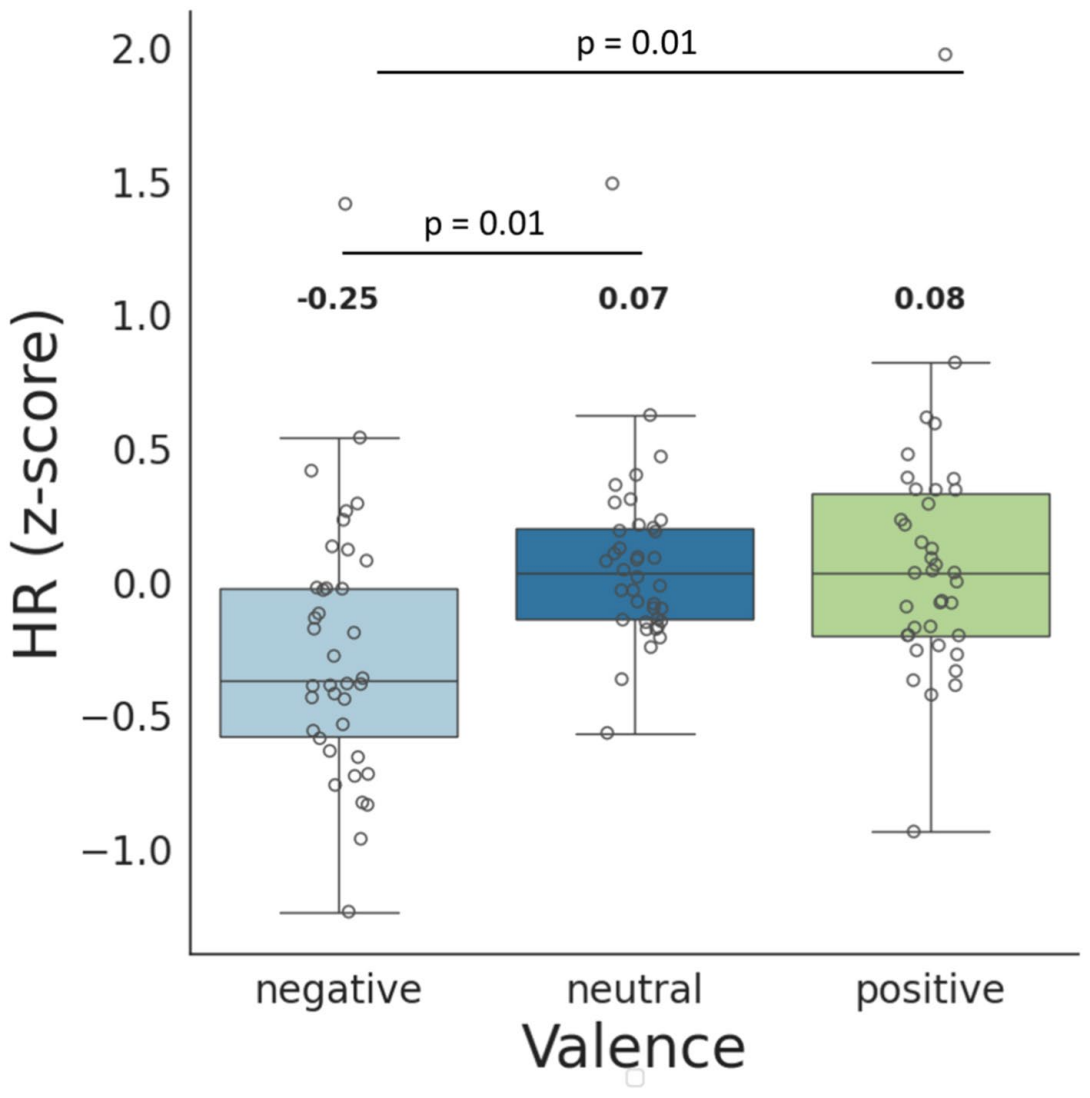
HR (*z*-score) when viewing negative, neutral, and positive videos. The box indicates the interquartile range (IQR), with the lower edge representing the 1st quartile (Q1) and the upper edge representing the 3rd quartile (Q3). The box contains the middle 50% of the data and medians, while the whiskers extend to the minimum and maximum values. Gray unfilled circles indicate individual data points. The indicated numbers represent the mean values for each group. The *p*-values for the *post-hoc* Tukey test are provided.

Estimated marginal means analysis revealed significantly lower heart rate z-scores during negative emotional valence compared to both neutral (estimate = −0.248, SE = 0.069, *p* < 0.001) and positive conditions (estimate = −0.254, SE = 0.077, *p* = 0.003). No significant difference was found between neutral and positive valence (*p* = 0.996) (Figure 3).

**Figure 4:**
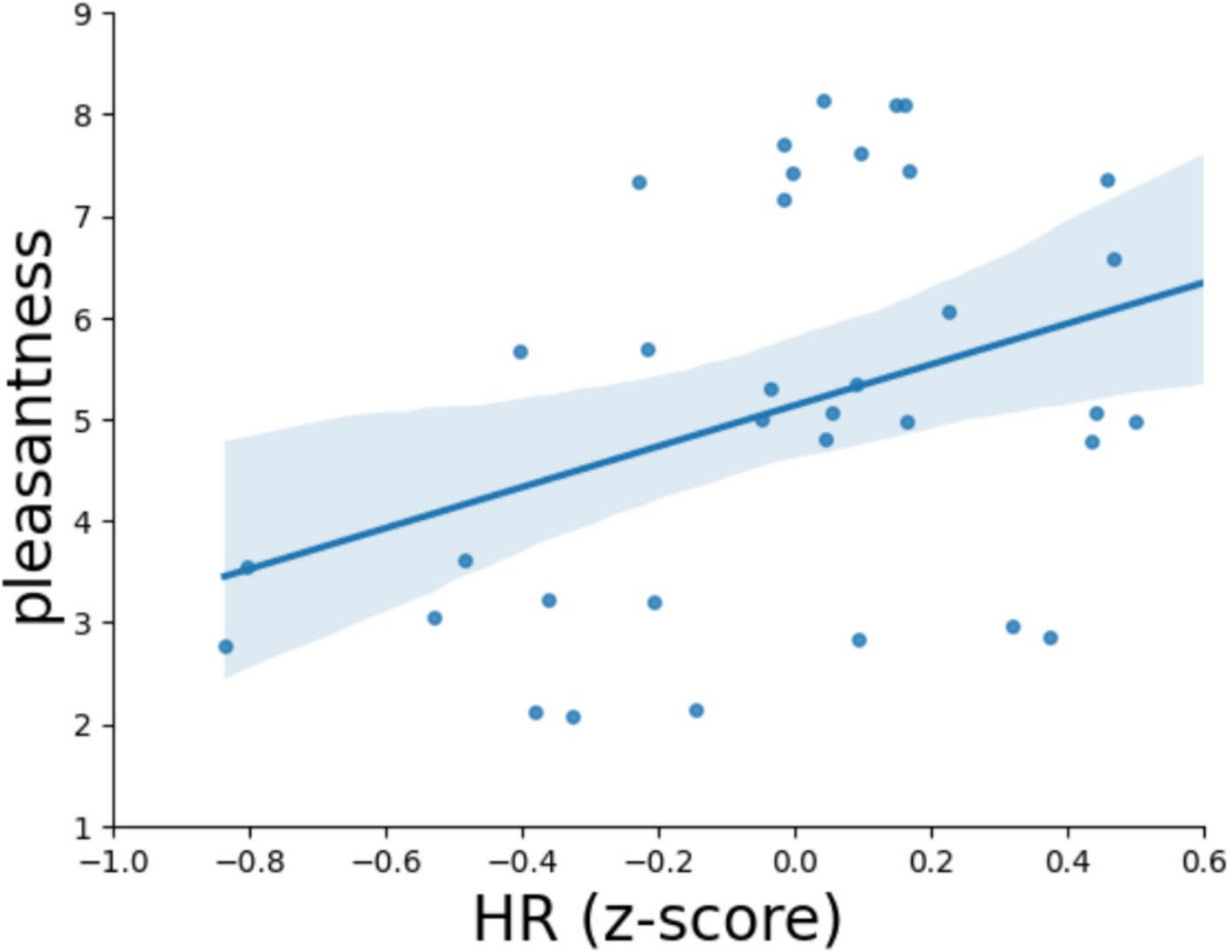
Correlation between the mean normalized heart rate of all subjects and the mean pleasantness scores of the video, *r* = 0.37, *p* = 0.014. One point corresponds to one video stimulus.

This is consistent with the results of the correlation analysis, which demonstrated a direct correlation between the subjective evaluation of video pleasantness averaged across all participants and the normalized HR observed while watching a given video, *r* = 0.37, *p* = 0.028. Thus, videos rated as unpleasant caused a slowing of the heart rate, while videos rated as pleasant, on the contrary, accelerated the HR (Figure 4).

### Heart rate and time perception

**Figure 5:**
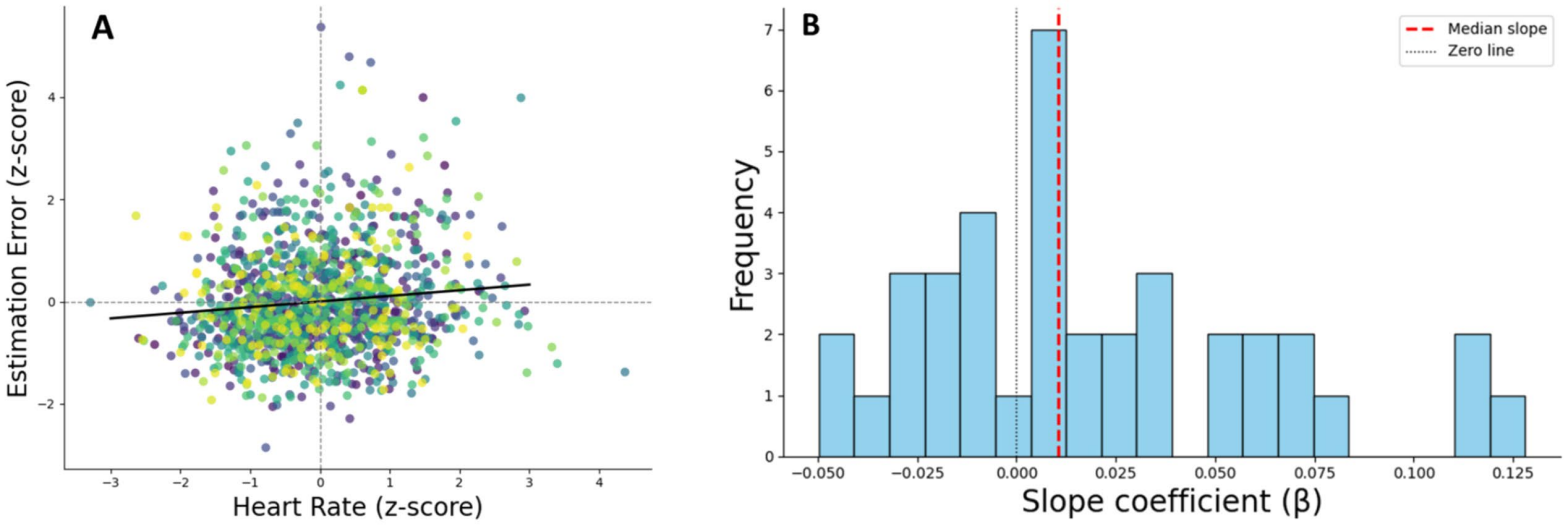
Individual relationships between heart rate and estimation error. **(A)** Scatterplot of *z*-scored heart rate (HR) against *z*-scored time estimation error, with individual participants shown in different colors. Each dot represents one trial. The black line indicates the group-level linear trend (*β* = −0.24), reflecting the overall association between HR and estimation error across participants. **(B)** Distribution of individual regression slopes from the relationship between heart rate (*z*-scored HR) and time estimation error across trials. Each slope represents the estimated change in estimation error per unit increase in *z*-scored HR for a single participant. The dashed red line indicates the median slope across all participants, while the dotted black line marks zero (no association). A predominance of negative slopes suggests that elevated heart rate was generally associated with underestimation of video duration.

In the next step, we examined the hypothesis concerning the relationship between heart rate and time perception. The mixed linear model regression analysis found that the normalized HR (*z*-score) factor was significantly associated with estimation error of video duration [*β* = 0.021, SE = 0.006, *z*-score = 3.592, *p* < 0.001, 97.5% CI (0.01, 0.033)]. Thus, the slower a person’s heart rate is, the less time they feel has passed (Figure 5).

When the data of only neutral videos were included in the model, the same pattern was observed, *β* = 0.021, SE = 0.009, *z*-score = 2.448, *p* < 0.014, 97.5% CI [0.001, 0.038], indicating that the effect of heart rate on time perception is independent or not fully dependent on emotional state.

### Heart rate as a mediator of emotional influence on time perception

To examine the hypothesis that heart rate mediates the influence of emotions on time perception, we conducted a Bayesian mediation analysis. The analysis revealed a significant indirect effect of emotions on time perception through HR. The estimated indirect effect (IE) was 0.0061, with a 94% highest density interval (HDI) of [0.0022, 0.0106], confirming that HR significantly mediates the relationship between emotions and temporal judgments. Emotional states exerted a strong influence on HR [*β* emotion_to_hr = 0.191, 94% HDI: (0.150, 0.238)], with negative emotions associated with a decrease in normalized HR. Normalized HR, in turn, had a significant impact on time perception errors [*β* hr_to_error = 0.032, 94% HDI: (0.013, 0.054)], such that slower heart rates were linked to greater underestimation of time intervals. The direct effect of emotions on time perception errors was more pronounced [*β* emotion_to_error = 0.072, 94% HDI: (0.049, 0.097)]. These findings support the hypothesis that HR serves as a mediator in the relationship between emotions and time perception, underscoring the importance of interoceptive signals in shaping temporal judgments. However, the indirect effect through HR was relatively weaker compared to the direct effect of emotions on time perception, suggesting that while physiological mechanisms play a role, psychological processes remain the dominant factor in shaping temporal judgments.

### Interoceptive accuracy and time perception

Other evidence in favor of the hypothesis that conscious perception of heartbeat can influence time perception would be the correlation of interoceptive accuracy and video duration estimation error. However, correlation analysis revealed no significant correlation between participants’ interoceptive accuracy and the participant’s average video duration error recorded during the experiment. It is important to note that the limited sample size (*n* = 38) provided sufficient power to detect medium to strong correlations, but may have been insufficient to identify weaker effects.

### Focus of attention, estimation error, and HR

The mixed linear model regression analysis with a random factor found that internal attention focus was associated with decreased estimation error of video duration [*β* = −0.026, SE = 0.012, *z*-score = −2.236, *p* = 0.025, 97.5% CI (−0.049, −0.003)] and decreased normalized heart rate [*β* = −0.176, SE = 0.056, *z*-score = −3.151, *p* = 0.002, 97.5% CI (−0.286, −0.067)]. However, there was no significant interaction between focus direction, normalized HR and pleasantness factors. Attention direction did not modulate the interplay between heart rate and time perception.

To validate the results obtained from the Linear Mixed Model, we conducted a Bayesian analysis. The analysis revealed that the effect of normalized heart rate on estimation error was small but statistically significant, with a posterior mean of 0.018 [94% HDI: (0.002, 0.035)]. This suggests that higher heart rates are associated with slightly increased estimation errors. Similarly, the effect of focus of attention (focus) was also significant, with a posterior mean of −0.022 [94% HDI: (−0.044, −0.001)], indicating that shifts in focus are associated with a slight reduction in estimation error. However, the interaction term between normalized HR and attention focus showed no significant effect, with a posterior mean of 0.003 [94% HDI: (−0.020, 0.027)], suggesting that the combined influence of these factors does not contribute meaningfully to estimation error. These results align with the LMM findings and support the use of a simpler model without the interaction term.

Descriptive statistics for *z*-score heart rate (HR) across different conditions are provided in Supplementary Table S5.

## Discussion

The present study aimed to investigate the hypothesis that interoceptive signals, particularly heart rate, mediate the influence of emotions on time estimation.

Our results confirmed that the perception of video duration can vary greatly depending on emotional content. Negative and neutral videos seemed shorter to participants than they actually were, while the estimation errors for positive videos were smaller. The impact of emotional state on time perception has been previously documented in several studies (Tipples, 2008; Yamada and Kawabe, 2011; Droit-Volet and Gil, 2009). However, the direction of this effect has varied among these studies. In some cases, similarly to ours, negative emotions resulted in an underestimation of the time intervals. Thus, in a study by Sarigiannidis et al. (2020a), induced anxiety led to underestimation of duration, thus, time seemed to pass quicker. Similarly, studies by Tamm et al. (2014) and Van Volkinburg and Balsam (2014) also found that negative emotions result in the underestimation of time intervals. Conversely, some studies reported an opposite effect. Anger and fear led to overestimation of the perception of time, and this effect was influenced by individual differences in negative emotionality (Tipples, 2008; Pollatos et al., 2014). Also, it was found that anxious people feel that time passes more slowly when they are exposed to short presentations of threatening stimuli (Bar-Haim et al., 2010). This discrepancy may stem from the various types and durations of stimuli presented, along with the different emotional nuances experienced by participants in the studies.

One of the previously proposed hypotheses for the underestimation of time during negative emotions is that individuals may misjudge the passage of time when their attention is directed inward, focusing on their internal experiences triggered by negative stimuli. Our findings support this hypothesis, demonstrating that an internal focus of attention is linked to an underestimation of time duration. There is an assumption that time passes more quickly by overloading neural resources, particularly in the mid-cingulate cortex, potentially driving emotion-related changes in temporal perception (Sarigiannidis et al., 2020b). Specifically, when a person experiences a strong emotion, their brain may become overloaded with processing that emotion, causing time to seem to pass faster.

Another hypothesis suggests that the physiological effects of emotions, such as fluctuations in heart rate, may also impact time perception. It is widely recognized that individuals experiencing negative emotions frequently exhibit an elevated heart rate (Wu et al., 2019; Karki and Mahara, 2022). However, our findings reveal a different phenomenon: watching videos that evoke negative emotions was associated with a decrease in heart rate, in contrast to videos featuring positive or neutral content. This phenomenon may be linked to the “freeze response,” a physiological reaction characterized by heightened attention and concentration. During this response, a decrease in heart rate can enhance our ability to detect and respond to potential threats, serving as a vital component of our stress-response system. This adaptive mechanism allows individuals to remain alert in potentially dangerous situations. Empirical evidence supports the notion that a slow heart rate can facilitate a heightened state of awareness. For instance, individuals displayed a freeze reaction alongside a decline in heart rate in response not only to physical threats but also in response to social threats, like angry faces (Roelofs et al., 2010; Noordewier et al., 2020). Additionally, responses to negative emotional stimuli often involve increased parasympathetic activity, which may lead to cardiac deceleration (Hagenaars et al., 2014). Research has shown that emotions such as disgust and sadness can activate the amygdala and are associated with this physiological response (Rohrmann et al., 2009). In certain circumstances, a reflex known as the vasovagal response may occur, characterized by simultaneous reductions in both heart rate and blood pressure in response to negative emotions (Dani et al., 2021). Such reactions can manifest in circumstances where an individual attempts to dissociate from their environment. When faced with some stressors, individuals may enter a state of “freezing,” which serves to minimize their visibility to potential dangers and facilitates a temporary emotional disengagement.

In future research, it would be highly beneficial to gather more detailed information from participants regarding the emotions they experience while watching videos. This information would be useful for drawing conclusions about the influence of specific types of positive and negative emotions on both physiological reactions and time perception.

In our study, we identified a significant association between heart rate and participants’ perception of time intervals. Our results showed that as heart rate decreased, participants tended to underestimate the duration of time intervals more significantly. This finding is consistent with a body of research conducted previously (Hawkes et al., 1961; Ogden et al., 2019). According to pacemaker–accumulator models, increased arousal or increased heart rate accelerates the internal clock, leading to time overestimation, while decreased arousal or heart rate deceleration may slow the pacemaker, resulting in temporal underestimation (Lake et al., 2016; Lediett and Tong, 1972). This relationship is not linear and can be influenced by additional factors such as emotional valence, attentional engagement, and interoceptive awareness. For example, Angrilli et al. (1997) demonstrated that time perception depends on a valence and arousal interaction: under low arousal, negative stimuli were perceived as shorter than positive ones, while under high arousal, negative stimuli were perceived as longer.

While the absence of subjective arousal ratings from participants during our experiment represents a limitation of the current study, pre-testing arousal ratings of stimuli confirmed that the negative and positive stimuli did not differ significantly in arousal levels, and both were rated as more arousing than neutral stimuli. Interestingly, we observed the strongest contrast in time estimation between positive and negative videos, but not between emotional (positive or negative) and neutral ones. This suggests that valence, rather than arousal alone, may be a more dominant factor in shaping temporal judgments in our paradigm.

Our results partially align with this: despite matched arousal levels between positive and negative stimuli, negative stimuli were still underestimated, suggesting that affective valence may exert an influence on timing independent of arousal level. This pattern could reflect the engagement of different motivational systems—approach vs. avoidance—or differing attentional demands associated with emotional valence, potentially mediated by distinct neural circuits. Importantly, based on pretested arousal ratings, the emotional stimuli used in our study fell within an intermediate range of arousal compared to those reported by Angrilli et al. (1997). Specifically, our negative stimuli showed arousal ratings between 5.63 and 6.40—a range that lies between the low-arousal negative stimuli (*M* = 4.0–5.7) and high-arousal negative stimuli (*M* = 6.5–7.5) identified in Angrilli et al.’s (1997) study.

Next, the Bayesian mediation analysis confirmed the study’s central hypothesis, demonstrating that heart rate (HR) partially mediates the effect of emotional valence on time estimation errors. While the contribution of the HR-mediated effect was found to be smaller compared to the impact of emotions through other mechanisms, not examined in this study, this finding underscores the intricate and multifaceted relationship between emotional states and temporal judgments. These results align with the idea that heartbeats, at least to some extent, act as a natural “time-keeper,” shaping our subjective experience of time. Indeed, the moment-to-moment perception of time appears to be closely synchronized with and dynamically influenced by the rhythm of the heartbeat.

Despite evidence suggesting that interoceptive signals play a role in time perception based on data regarding the relationship between heart rate and perception of time (Meissner and Wittmann, 2011; Khoshnoud et al., 2024), our analysis of the entire sample revealed no correlation between participants’ interoceptive accuracy and their mean duration estimation error. This contrasts with some previous studies that identified such a relationship (Uraguchi et al., 2022; Teghil et al., 2020). These discrepancies may be attributed to differences in study design and the specific tasks used to assess time perception. In the study by Uraguchi et al. (2022), participants were required to compare the durations of two stimuli, whereas in Teghil et al. (2020), participants had to reproduce the duration of a sample stimulus. These variations in task demands could influence the extent to which interoceptive signals contribute to temporal judgments. Additionally, it is important to note that Teghil and colleagues assessed interoceptive awareness using a self-report questionnaire, which captures subjective perceptions of interoception rather than objective measures of interoceptive accuracy, as was employed in our study. Such methodological differences likely contribute to the divergent findings. Furthermore, the relatively small sample size of our study (38 participants) may have limited our ability to detect weaker correlations between interoceptive accuracy and time perception errors, highlighting a potential constraint in drawing definitive conclusions from our results.

An inward focus of attention was associated with a more pronounced underestimation of video duration and a lower normalized heart rate. However, we did not observe any significant interactions between focus direction, normalized heart rate, and emotion valence, unlike study by Pollatos et al. (2014), which indicated that an internal focus of attention amplifies the influence of emotional state on duration estimation error. One possible explanation for this finding is that, as our results demonstrated, heart rate (HR) only partially mediates the effect of emotions on time perception, with its contribution being smaller compared to other mechanisms that were not explored in this study. This limited mediating role of HR may explain why modulating attention toward internal signals did not reveal a significant effect, as the influence of attention might be outweighed by other, more dominant pathways through which emotions impact temporal judgments. Additionally, another potential reason could be the involvement of subconscious interoceptive processes that are unaffected by attentional focus. These underlying mechanisms may play a significant role in shaping time perception independently of whether attention is directed inward or outward, thus contributing to the absence of observed interactions in our study.

The differences between our findings and those reported by Pollatos et al. (2014) are particularly important to discuss, as our study was directly inspired by this work and aimed to extend it. In their study, it was demonstrated that an interoceptive focus amplifies the influence of emotional states on duration perception for 40-s video clips. Specifically, they found that amusing videos were underestimated in duration, while fearful videos were overestimated, with the internal focus enhancing these emotion-driven temporal distortions.

In contrast, our study revealed different patterns: positive videos tended to be overestimated, negative videos were underestimated, and we did not observe any significant interaction between focus direction (internal vs. external) and emotional valence in modulating duration estimation errors. These discrepancies warrant a closer examination, particularly given the methodological similarities between the two studies. One possible explanation for the differing results lies in the nature of the stimuli used. Pollatos et al. (2014) employed a limited set of three stimuli (one fear-inducing, one neutral, and one engaging video) which allowed for a focused exploration of specific emotions but restricted the generalizability of their findings. In our study, we adopted a broader design, presenting participants with 36 diverse video clips selected from a pretested database to elicit a wide range of emotional responses. Additionally, we incorporated subjective ratings provided by participants during the experiment to account for individual variability in emotional experiences. While this approach enhances the generalizability of our findings regarding the interaction between attentional focus and emotional valence, it may also introduce variability that obscures the specific effects of discrete emotions. In summary, while our study builds on the foundational insights of Pollatos et al. (2014), the contrasting results highlight the critical role of stimulus selection and methodological design in shaping outcomes. The use of a broader stimulus set and the inclusion of physiological measures in our study offer valuable new perspectives on the interplay between attentional focus, emotional valence, and time perception. However, these differences also underscore the need for further research to reconcile the findings and explore how specific emotions, individual differences, and physiological factors interact to shape temporal judgments.

In conclusion, our study provides valuable insights into the intricate relationships between emotions, heart rate, and time perception. We demonstrated that heart rate partially mediates the influence of emotions on temporal judgments, supporting the hypothesis that interoceptive signals play a crucial role in shaping our subjective experience of time. However, the relatively small contribution of the heart rate-mediated effect compared to other mechanisms highlights the multifaceted nature of time perception. Additionally, our findings suggest that attentional focus does not significantly modulate the relationship between emotional valence and time perception, pointing to the potential dominance of subconscious interoceptive processes over conscious awareness. These results emphasize the importance of considering both physiological and psychological factors when exploring temporal judgments. Understanding these complex interactions not only deepens our knowledge of time perception but also holds promise for addressing challenges related to emotional well-being and body perception, ultimately fostering more integrated self-representations and temporal awareness.

## Data Availability

The original contributions presented in the study are included in the article/Supplementary material, further inquiries can be directed to the corresponding author.
