# Supplementary material for: Interoceptive signals and emotional states shape temporal perception through heart rate modulation
Source: Front Psychol. 2025 Jul 3;16:1610347. doi: 10.3389/fpsyg.2025.1610347 (PMC12267279; doi:10.3389/fpsyg.2025.1610347)

# Descriptive statistics for z-score heart rate (HR) across different conditions

Table 1. Mean values of z-score HR and estimation error (± standard deviation) for different focus conditions. Values represent the mean (± standard deviation) of z-score HR and estimation error, averaged across participants for each combination of emotion and focus condition (negative, neutral, positive). Data were pre-processed by averaging individual participant scores within each emotion-focus group prior to calculating overall means.


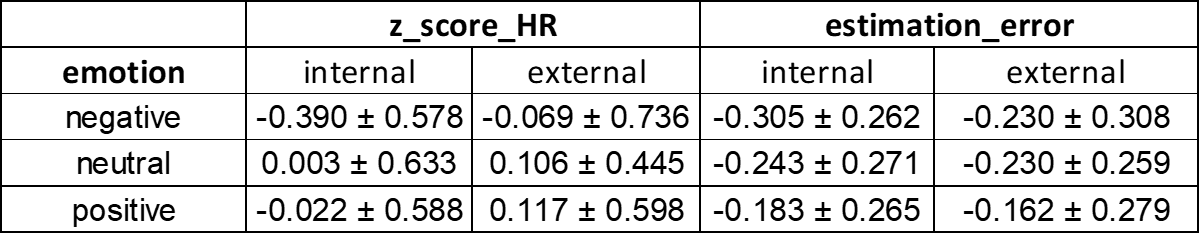

Supplement: Supplementary file 3 [file Table_3.DOCX]
