# Supplementary material for: Interoceptive signals and emotional states shape temporal perception through heart rate modulation
Source: Front Psychol. 2025 Jul 3;16:1610347. doi: 10.3389/fpsyg.2025.1610347 (PMC12267279; doi:10.3389/fpsyg.2025.1610347)
Supplement: Supplementary file 4 [file Table_4.DOCX]

**Description of statistical models**

This document provides a detailed description of all statistical models used in the study. The analysis was conducted using Python, with a combination of linear mixed models (LMMs) implemented via `statsmodels` and Bayesian hierarchical models implemented via `PyMC`. Below is a structured explanation of each model, including its purpose, implementation, and key parameters.

## 1. Linear Mixed Models (LMMs)

Linear mixed models (LMMs) were employed to evaluate relationships between key variables while accounting for individual differences by incorporating participant ID as a random factor. The `statsmodels.formula.api.mixedlm` function was used for this analysis. Below are the specific models implemented:

**Model 1: Relationship Between Estimation Error and Video Pleasantness**

md = smf.mixedlm("estimation_error ~ est_like", data, groups=data["subj_id"])

- Purpose: To assess the relationship between subjective video pleasantness (`est_like`) and time perception errors (`estimation_error`).

- Fixed Effects: `est_like` (subjective pleasantness rating of the video on a 9-point scale).

- Random Effects: Random intercepts for participants (`subj_id`).

**Model 2: Relationship Between Estimation Error and Normalized Heart Rate**

md = smf.mixedlm("estimation_error ~ z_score_HR", data, groups=data["subj_id"])

- Purpose: To examine the association between normalized heart rate (`z_score_HR`) and time perception errors (`estimation_error`).

- Fixed Effects: `z_score_HR`.

- Random Effects: Random intercepts for participants (`subj_id`).

**Model 3: Relationship Between Normalized Heart Rate and Video Pleasantness**

md = smf.mixedlm("z_score_HR ~ est_like", data, groups=data["subj_id"])

- Purpose: To explore how subjective video pleasantness (`est_like`) influences normalized heart rate (`z_score_HR`).

- Fxed Effects: `est_like`.

- Random Effects: Random intercepts for participants (`subj_id`).

**Model 4: Interaction Between Normalized Heart Rate and Focus of Attention**

md = smf.mixedlm("estimation_error ~ z_score_HR * C(focus)", data, groups=data["subj_id"], re_formula='~ interoceptive_accuracy')

- Purpose: To test whether focus of attention interacts with normalized heart rate (`z_score_HR`) to influence time perception errors (`estimation_error`).

- Fixed Effects: Interaction term between `z_score_HR` and `focus` (coded as binary: internal, external).

- Random Effects: Random intercepts for participants (`subj_id`) with random slopes for interoceptive accuracy.

## 2. Bayesian Hierarchical Analysis

To validate findings from the LMMs and explore specific hypotheses, we conducted a Bayesian hierarchical analysis using the PyMC library. This analysis incorporated random intercepts for participants to account for within-subject variability and estimated fixed effects for normalized heart rate (`z_score_HR`), focus of attention, and their interaction.

Data Preparation

data["focus"] = data["focus"].map({"internal": 1, "external": 0})

unique_subjects = data["subj_id"].unique()
subject_idx = data["subj_id"].astype("category").cat.codes.values

Bayesian Model Specification

with pm.Model() as model:
 # Hyperparameters for random effects
 mu_alpha = pm.Normal("mu_alpha", mu=0, sigma=1) # Mean for random intercepts
 sigma_alpha = pm.HalfNormal("sigma_alpha", sigma=1) # Variance for random intercepts
 alpha = pm.Normal("alpha", mu=mu_alpha, sigma=sigma_alpha, shape=len(unique_subjects)) # Random intercepts

 # Fixed effects
 beta_hr = pm.Normal("beta_hr", mu=0, sigma=1) # Coefficient for z_score_HR
 beta_focus = pm.Normal("beta_focus", mu=0, sigma=1) # Coefficient for focus
 beta_interaction = pm.Normal("beta_interaction", mu=0, sigma=1) # Coefficient for interaction

 # Linear model
 mu = (
 alpha[subject_idx] +
 beta_hr * data["z_score_HR"] +
 beta_focus * data["focus"] +
 beta_interaction * data["z_score_HR"] * data["focus"]
 )

 # Residual variance
 sigma = pm.HalfNormal("sigma", sigma=1)

 # Likelihood
 likelihood = pm.Normal("likelihood", mu=mu, sigma=sigma, observed=data["estimation_error"])

 # Posterior sampling
 trace = pm.sample(2000, tune=1000, random_seed=42, cores=2)

## 3. Bayesian Mediation Analysis

To test the hypothesis that heart rate mediates the effects of emotions on time perception, we performed a Bayesian mediation analysis using a hierarchical linear mixed-effects model. The model included two pathways:

- Path A: Emotions → Normalized HR

- Path B: Normalized HR → Time Perception Error

Mediation Model

import pymc as pm
import arviz as az

with pm.Model() as mediation_model:
 mu_alpha = pm.Normal("mu_alpha", mu=0, sigma=1)
 sigma_alpha = pm.HalfNormal("sigma_alpha", sigma=1)
 alpha = pm.Normal("alpha", mu=mu_alpha, sigma=sigma_alpha, shape=len(data["subj_id"].unique()))


 beta_emotion_to_hr = pm.Normal("beta_emotion_to_hr", mu=0, sigma=1)
 beta_hr_to_error = pm.Normal("beta_hr_to_error", mu=0, sigma=1)
 beta_emotion_to_error = pm.Normal("beta_emotion_to_error", mu=0, sigma=1)
 mu_hr = alpha[data["subj_id"].cat.codes] + beta_emotion_to_hr * data["emotion_numeric"].values
 mu_error = (
 alpha[data["subj_id"].cat.codes] +
 beta_hr_to_error * data["z_score_HR"].values +
 beta_emotion_to_error * data["emotion_numeric"].values
 )

 sigma_hr = pm.HalfNormal("sigma_hr", sigma=1)
 sigma_error = pm.HalfNormal("sigma_error", sigma=1)
 hr_likelihood = pm.Normal("hr_likelihood", mu=mu_hr, sigma=sigma_hr, observed=data["z_score_HR"].values)
 error_likelihood = pm.Normal("error_likelihood", mu=mu_error, sigma=sigma_error, observed=data["estimation_error"].values)
 trace = pm.sample(2000, tune=1000, random_seed=42, cores=2)

Key Components

- Random Effects: Random intercepts for participants.

- Fixed Effects:

- `beta_emotion_to_hr`: Effect of emotions on normalized HR.

- `beta_hr_to_error`: Effect of normalized HR on time perception errors.

- `beta_emotion_to_error`: Direct effect of emotions on time perception errors.

- Indirect Effect: Calculated as the product of `beta_emotion_to_hr` and `beta_hr_to_error`.

## 4. Rationale for the chosen statistical approaches

**Linear Mixed Models (LMMs):**

We employed LMMs to examine the relationships between emotional valence, heart rate (HR), time estimation error, and focus of attention. This choice was motivated by the hierarchical nature of our data, where multiple observations (video trials) were nested within individual participants. LMMs allow us to account for this within-subject dependency by including random intercepts for participant ID, thus controlling for individual variability in baseline estimation error or heart rate responses. Furthermore, LMMs are robust to unbalanced data, which is common in psychological experiments due to missing responses or trial exclusions.

**Estimated Marginal Means (EMMs):**

To explore pairwise differences between emotion categories, we used EMMs based on subjective pleasantness ratings. This approach allowed us to estimate the average effect of each emotion category while adjusting for other factors in the model, and to make multiple comparisons with appropriate corrections (Tukey’s adjustment), enhancing the interpretability and validity of our post hoc contrasts.

**Bayesian Hierarchical Modeling (PyMC):**

We conducted Bayesian analyses to verify and quantify the absence of interaction effects, particularly between HR and focus of attention. This approach is advantageous in that it allows for direct probability statements about parameters, rather than relying on p-values alone, and is particularly informative when evaluating evidence for the absence of an effect. The hierarchical structure, including random intercepts for subjects, preserved consistency with our previous models and ensured that individual variability was still properly accounted for.

**Bayesian Mediation Analysis:**

The central hypothesis of our study proposed that heart rate mediates the relationship between emotions and time perception. A Bayesian hierarchical mediation model allowed us to test both mediation paths (A and B) while incorporating random effects for participants. This approach is particularly suited for estimating indirect effects in multilevel designs and provides credible intervals for the mediated effect, which is more informative and statistically appropriate in small-to-moderate samples compared to traditional frequentist mediation models.

**Pearson Correlation Analysis:**

For exploring individual differences, such as the association between interoceptive accuracy and average estimation error, or the correlation between mean HR and pleasantness ratings, we used Pearson correlations. These simple bivariate analyses complemented our main models by examining subject-level summary metrics. While more limited in scope, they offered intuitive insights and served as confirmatory or exploratory tools in line with prior literature.

Overall, each statistical technique was selected to match the level of data aggregation, the dependency structure, and the specific hypothesis being tested. Frequentist mixed models were used to examine trial-level effects across the full dataset, while Bayesian models were applied when estimating interactions or indirect effects where probabilistic interpretation and model flexibility were beneficial.
