# Supplementary material for: Interoceptive signals and emotional states shape temporal perception through heart rate modulation
Source: Front Psychol. 2025 Jul 3;16:1610347. doi: 10.3389/fpsyg.2025.1610347 (PMC12267279; doi:10.3389/fpsyg.2025.1610347)
Supplement: Supplementary file 5 [file Table_5.DOCX]

**Arousal level and duration of videos categorized by subjective ratings**

To characterize the properties of the video stimuli used in the study, we analyzed the mean duration and arousal level of videos categorized as negative, neutral, and positive. Arousal ratings were based on pre-tested values obtained during stimulus validation.

As expected based on pre-test ratings, negative and positive valence videos were characterized by high arousal levels (negative: M = 5.85, SD = 0.20; positive: M = 5.83, SD = 0.22). In contrast, neutral videos had significantly lower arousal (M = 4.53, SD = 0.46). These findings were confirmed by estimated marginal means analysis with pairwise comparisons: negative and positive valence conditions did not differ in arousal (estimate = 0.003, SE = 0.076, p = 0.999), whereas both differed significantly from neutral (negative vs. neutral: estimate = 1.151, SE = 0.068, p < .0001; neutral vs. positive: estimate = –1.148, SE = 0.066, p < .0001).

Negative videos had an average duration of 39.98 seconds (SD = 4.41), and positive videos lasted slightly longer at 41.03 seconds (SD = 2.59). Neutral videos were somewhat shorter, with a mean of 37.06 seconds (SD = 2.00). A pairwise comparison using estimated marginal means revealed only a trend to significant difference between negative and neutral conditions (estimate = 1.89, SE = 0.824, p = 0.056). No significant difference was found between negative and positive videos (estimate = –1.76, SE = 0.916, p = 0.133). Neutral videos were significantly shorter than positive videos (estimate = –3.65, SE = 0.797, p < .0001).
